# Antiviral and Immunomodulatory Properties of Antimicrobial Peptides Produced by Human Keratinocytes

**DOI:** 10.3389/fmicb.2020.01155

**Published:** 2020-06-03

**Authors:** Céline Chessa, Charles Bodet, Clément Jousselin, Michel Wehbe, Nicolas Lévêque, Magali Garcia

**Affiliations:** ^1^Laboratoire de Virologie et Mycobactériologie, CHU de Poitiers, Poitiers, France; ^2^Laboratoire Inflammation, Tissus Epithéliaux et Cytokines, LITEC EA 4331, Université de Poitiers, Poitiers, France

**Keywords:** keratinocyte, antimicrobial peptide, innate immunity, antiviral, immunomodulation

## Abstract

Keratinocytes, the main cells of the epidermis, are the first site of replication as well as the first line of defense against many viruses such as arboviruses, enteroviruses, herpes viruses, human papillomaviruses, or vaccinia virus. During viral replication, these cells can sense virus associated molecular patterns leading to the initiation of an innate immune response composed of pro-inflammatory cytokines, chemokines, and antimicrobial peptides. Human keratinocytes produce and secrete at least nine antimicrobial peptides: human cathelicidin LL-37, types 1–4 human β-defensins, S100 peptides such as psoriasin (S100A7), calprotectin (S100A8/9) and koebnerisin (S100A15), and RNase 7. These peptides can exert direct antiviral effects on the viral particle or its replication cycle, and indirect antiviral activity, by modulating the host immune response. The purpose of this review is to summarize current knowledge of antiviral and immunomodulatory properties of human keratinocyte antimicrobial peptides.

## Introduction

As the largest organ of human body, skin is not only a physical barrier but represents also the first line of defense against environmental pathogens including viruses (Robert and Kupper, 1999; Ganz, 2002). Skin is organized in three layers, which differ structurally and functionally: epidermis, the most superficial, dermis and hypodermis, the deepest. The epidermis is mainly composed of keratinocytes at different levels of differentiation, from the stratum basale made of the youngest keratinocytes, still dividing, at the dermis interface to the keratin containing desquamating corneocytes on the surface of the skin. Langerhans cells (LCs), a skin-specialized type of dendritic cells (DCs) constantly probing for antigens, represent the main immune resident cell in the epidermis (Kubo et al., 2009). T cells, mainly CD8+, can also be found in the deepest strata, stratum basale, and stratum spinosum, of the epidermis (Nestle et al., 2009). Finally, melanocytes, responsible for the pigmentation of the skin, constitute the last cell type of the epidermis (Nestle et al., 2009). Then, the dermis is a more complex conjunctive tissue composed of several specialized cells, such as DCs, CD4 + T helper (Th)1, Th2, and Th17 cells, γδ T cells, macrophages, mast cells, and fibroblasts, which all together play a role in the immune skin barrier. Contrary to the epidermis, the dermis is an innervated tissue where blood and lymphatic vessels contribute to cell trafficking (Nestle et al., 2009). Finally, hypodermis is an adipose-tissue mainly composed of fat cells.

Keratinocytes allow, *in vivo* and *in vitro*, replication of many viruses such as alphaherpesviruses [types 1 and 2 herpes simplex viruses (HSV), varicella-zoster virus (VZV)], arboviruses such as Dengue (DENV), Zika (ZIKV), and West Nile viruses (WNV), enteroviruses, human papillomaviruses (HPVs) and vaccinia virus (VACV), amplifying the viral load and facilitating viral spread to the liver, the fetus or the central nervous system (Lim et al., 2011; Crack et al., 2012; Puiprom et al., 2013; Sayers and Elliott, 2016**;** Gourru-Lesimple et al., 2017; Phyu et al., 2017; Duangkhae et al., 2018). Keratinocytes also act as immune cells that can initiate an innate immune response to fight viral infection (Nestle et al., 2009). Indeed, they express a wide range of Pattern-Recognition Receptors (PRRs) including cell-surface (-1, -2, -4, -5, -6) or endosomal (-3, -7, -8, -9) transmembranal Toll-Like Receptors (TLRs). They also possess cytosolic sensors like Retinoic acid Inducible Gene I (RIG-I)-Like Receptors (RLRs): Melanoderma Differentiation-Associated gene 5 (MDA5), Retinoic acid-Inducible gene (RIG-I) and Laboratory of Genetics and Physiology 2 (LGP2), or cytosolic DNA receptor cyclic GMP-AMP Synthase (cGAS) (Almine et al., 2017; Nestle et al., 2009). All these PRRs sense Pathogen Associated Molecular Patterns (PAMPs) that are, in case of viruses, nucleic acid and structural or non-structural protein motifs conserved among pathogens (Yong and Luo, 2018). For RNA viruses, such as arboviruses and enteroviruses, the main PRRs involved in the detection of the viral infection are TLRs 3 and 7, and RLRs RIG-I and MDA5, detecting single-stranded viral genomes or double-stranded replication intermediates in endosomes and cytoplasm, respectively. Activation of these PRRs triggers signaling pathways leading to production of interferons (IFNs), proinflammatory chemokines, cytokines and antimicrobial peptides (AMPs) that are key players of the innate immune response Kalali et al., 2008; Nestle et al., 2009; Takeuchi and Akira, 2009; Welte et al., 2009**;** Aguirre et al., 2012; Dalpke and Helm, 2012; Garcia et al., 2017). Then, secreted pro-inflammatory mediators participate to the recruitment of monocytes, macrophages, polymorphonuclear neutrophils (PMNs), and DCs to the site of viral infection (Schmid and Harris, 2014; Pingen et al., 2016; Sharif et al., 2016). These cells are innate immune sentinels playing a crucial role in activation of innate and adaptive antiviral immunities. For example, once activated by a viral antigen, LCs and DCs start migrating to the draining lymph node in order to prime T-cells and induce an immune memory (Johnston et al., 1996, 2000; Kubo et al., 2009). Other skin cells can also contribute to the initiation of the innate immune response. It has been suggested that melanocytes were contributing to the phagocytosis of viral pathogens, presenting antigens to competent immune cells or producing cytokines and chemokines (Gasque and Jaffar-Bandjee, 2015). Overall, these data highlight the crosstalk between the different kinds of skin cells in order to create an antiviral environment. Thus, in the same way as respiratory, genital or digestive epithelia, the cutaneous epithelium is an interface between the organism and the outside environment exposed to many microorganisms, functioning as a physical but also as an immunological barrier (Grice and Segre, 2011). Antimicrobial defense is therefore essential in order to preserve the “aseptic” of deep skin tissue. For this purpose, AMPs, which are small peptides synthesized and secreted by skin cells and glands, display antifungal, antibacterial and both direct and indirect antiviral activities (Zasloff, 2002). Indeed, they can directly inactivate viral particles or inhibit virus replication (Wilson et al., 2013). AMPs can also exert an indirect antiviral activity by modulating the host immune response. They can induce the production of cytokines and chemokines, demonstrating both proper pro-inflammatory activities and potentiating the inflammatory response caused by the infection. AMPs may also exert a chemo-attractant activity on immune cells at the site of infection contributing to viral clearance (De et al., 2000; Koczulla and Bals, 2003; Tjabringa et al., 2006; Lai and Gallo, 2009; Mookherjee et al., 2009).

The purpose of this review is to summarize current knowledge about direct antiviral and immunomodulatory properties of human keratinocyte antimicrobial peptides.

## Main Characteristics of Antimicrobial Peptides Produced by Human Keratinocytes and Expression in the Context of Viral Infection

Antimicrobial peptides are small peptides, classically less than 100 amino-acids, with a large structural diversity (Lehrer and Ganz, 1999; Lei et al., 2019). They can form α-helices, cysteine-rich pleated β-sheets with one or more disulfide bridges, or be relatively non-structured peptides containing a high percentage of one specific type of amino-acid (Lehrer and Ganz, 1999). Human keratinocytes are known to produce and secrete at least nine AMPs: human cathelicidin LL-37, types 1–4 human β-defensins, psoriasin (S100A7), calprotectin (S100A8/9), koebnerisin (S100A15), and RNase 7 (Braff et al., 2005; Lai and Gallo, 2009). Their expression can be constitutive or inducible, displaying an increased expression in case of stimuli such as infection or inflammation. In addition, incubation of primary human keratinocytes with pro-inflammatory cytokines can stimulate AMP synthesis (Guilloteau et al., 2010; Firat et al., 2014).

### Human Cathelicidin LL-37

LL-37 is the only known human cathelicidin, although peptides of this large family have been isolated from numerous non-human species. This amphipathic α-helical peptide is produced by human keratinocytes during inflammatory disorders like psoriasis, lupus erythematosus and contact dermatitis where its concentration can reach 20 μg/mL (Frohm et al., 1997; Braff et al., 2005; Currie et al., 2013). Its up-regulation has also been described during HSV-2 and DENV infections of human keratinocytes (Ogawa et al., 2013; López-González et al., 2018). Moreover, LL-37 expression in combination with hBD-3 was also increased in epidermal and dermal lesions of patients suffering from Kaposi’s sarcoma caused by human herpes virus 8 (HHV-8) in comparison to normal skin of healthy controls (Fathy et al., 2012). Finally, the secretion of LL-37 was observed during the infection of the respiratory epithelium by human rhinovirus (HRV) C, influenza virus A (IAV)/H1N1 and respiratory syncytial virus (RSV) (Hansdottir et al., 2008; Boda et al., 2018). In cell culture supernatant of infected nasal epithelium, LL-37 secretion ranged from 10 to 25 ng/mL. Interestingly, its secretion was not detected following HRV-B and coronavirus (HCoV-) OC43 infection potentially related to a different cell tropism of these two viral species (Boda et al., 2018).

### β-defensins

Defensins are small molecules between 24 and 42 amino-acids characterized by a β-sheet structure with 3 disulfide bounds. In human, defensins are divided into α-defensins, referred to as human neutrophil peptides (hNPs), and β-defensins (hBDs) expressed in myeloid and epithelial cells. There are about 37 hBDs (Wilson et al., 1999; Holly et al., 2017). Four (hBD-1 to -4) are detected in the epidermis. Many viruses were shown to stimulate hBD expression and/or secretion in epithelial cells, even though the antiviral activity of these peptides was not always demonstrated (Frohm et al., 1997). In human keratinocytes, VACV, DENV and HSV-2 infections were shown to induce hBD-1, hBD-2, and -3, and hBD-4 expression, respectively (reviewed in Surasombatpattana et al., 2011; Wilson et al., 2013). HPV infection also increased hBD-1 to -3 expression in oral epithelial lesions from patients with recurrent respiratory papillomatosis (Chong et al., 2006). hBD production was then demonstrated in vulvovaginal biopsy samples of condylomata acuminata as well as in human amniotic epithelial cells infected with HPV (Erhart et al., 2011; Szukiewicz et al., 2016). Interestingly, expression of hBD-2 was paradoxically diminished in HPV-induced carcinomas potentially defining a mechanism of virus escape to the host immune response occurring during carcinogenesis (Hubert et al., 2007). Type 1 human immunodeficiency virus (HIV-1) infection also induced expression of hBD-2 and -3, but not that of hBD-1, in human oral epithelial cells (Quiñones-Mateu et al., 2003). In respiratory epithelial cells infected *in vitro*, hBD-2 and hBD-3 production is stimulated by the replication of several HRV serotypes from HRV-B and -C species. *In vivo*, a doubling of the concentration of hBD2, from 150 ng/mL to more than 300, was assessed at 48 h post-infection in nasal swabs of patients infected with HRV-A16 (Proud et al., 2004). Similarly, IAV/H1N1 as well as RSV infections induced a huge increase in hBD-2 secretion whereas HCoV-OC43 did not (Kota et al., 2008; Boda et al., 2018). In intestinal epithelial cells, enterovirus (EV) infection enhanced the secretion of hBD-3 but not that of α- and other β-defensins (Chen et al., 2018). In fresh peripheral blood mononuclear cells, the other major source of hBD production in human, only hBD-1 coding mRNAs were detected in non-stimulated cells among the four known hBDs (Oppenheim et al., 2003). Its expression could be then induced as early as 3 h post-infection with IAV, Sendai virus or, in a much lesser extent, HSV-1 (Ryan et al., 2011). Finally, hBD concentrations have been demonstrated to be elevated after exposure to Hepatitis B (HBV) and C (HCV) viruses as well as to Crimean-Congo hemorrhagic fever virus (CCHFV) (Bai et al., 2015; Aksoy et al., 2016; Mattar et al., 2016a). Concentrations of hBDs were shown to be significantly higher in HCV-infected patient sera, ranging from 900 to 21,120 ng/mL, compared to controls where they were less than 60 ng/mL (Mattar et al., 2016a). In the same way, serum hBD2 levels were significantly increased in patients infected with CCHFV compared to healthy controls and were three-times higher in patients with non-fatal evolution of the disease than in patients with fatal disease (89,480 vs. 30,580 ng/mL) suggesting a protective role of the peptide during the infection (Aksoy et al., 2016).

### Peptides of the S100 Family

S100 family peptides are characterized by two calcium-binding sites that can also chelate zinc and manganese. This family regroups 25 molecules among those psoriasin S100A7, calprotectin S100A8/9 and koebnerisin S100A15 are produced by keratinocytes (Celis et al., 1990; Zhu et al., 2013). Constitutive expression of psoriasin S100A7 is low in normal adult skin ranging from 5 to 46 ng/cm^2^ regarding to the region of the human body (Gläser et al., 2005). Its expression can be enhanced in stimulated keratinocytes as seen in psoriasis (Gläser et al., 2005). Conversely, high expression levels have been detected in the fetal skin, suggesting a potentially protective role in the innate immune system of the newborn (Yoshio et al., 2003). Besides its antimicrobial activity, S100A7 is associated with wound healing, neutrophil migration, Reactive Oxygen Species (ROS) generation, antimicrobial peptide release and cytokine/chemokine production (Niyonsaba et al., 2008; Kozlyuk et al., 2019). S100A7 has further been reported to be overexpressed in breast and bladder tumors, suggesting that it may play a role in the regulation of cell growth, survival and differentiation (Watson et al., 1998; Ostergaard et al., 1999). Regarding viral infections, psoriasin expression is induced in vulvovaginal and cervical HPV-associated lesions (Erhart et al., 2011; Alvendal et al., 2019). Calprotectin S100A8/9 is a heterodimer composed of calgranuline A (S100A8 or myeloid-related protein 8) and calgranuline B [S100A9 or migration inhibitory factor-related protein 14 (MRP14)]. In normal epidermis, S100A8 and S100A9 are both expressed at low levels but, in inflammatory skin diseases such as psoriasis, lichen planus and lupus erythematosus, or during wound healing, their expression is highly induced (Gabrielsen et al., 1986; Kunz et al., 1992; Thorey et al., 2001). Moreover, S100A8/9 expression has been identified as a general danger signature of activated keratinocytes, as its expression can be induced in response to a wide variety of skin stresses including tape stripping, exposure to detergent, UV or cytokine stimulation (IL-1α, IL-22) (Boniface et al., 2005; Ehrchen et al., 2009). This overexpression prevents keratinocyte proliferation but triggers cell differentiation (Ryckman et al., 2003; Voss et al., 2011). Calprotectin expression has been also shown to be increased in epithelial cells during viral infections by coronavirus and HPV with antiviral properties against HPV type 16 (Reghunathan et al., 2005; Tugizov et al., 2005). Finally, koebnerisin S100A15, which has a sequence almost identical to that of psoriasin, is overexpressed in psoriatic skin lesions and known for its proinflammatory and chemotaxis properties (Wolf et al., 2011). Infection with *Escherichia coli* also modulates its expression in keratinocytes through recognition of the pathogen by TLR4 (Büchau et al., 2007). To our knowledge, S100A15 expression in the context of viral infection has so far never been studied.

### RNase 7

While RNase 7 is usually considered as an AMP, it is actually a larger protein of 14.5 kDa, composed of 128 amino acids and belonging to the RNase A superfamily. RNase 7 exhibits potent ribonuclease activity and its expression in the skin is both constitutive and inducible in inflammatory and infectious contexts (Harder and Schroder, 2002; Simanski et al., 2013; Firat et al., 2014). In normal skin, RNase 7 concentration varies according to the area of the body, from 0.17 ng/cm^2^, in the palms of the hands, to 1.28 ng/cm^2^, in skin of the legs (Rademacher et al., 2016). RNase 7 concentrations are increased in patients with psoriatic, atopic dermatitis and dermatophyte skin lesions (Becknell and Spencer, 2016). *In vitro* studies have demonstrated that the treatment of primary keratinocytes with proinflammatory cytokines such as IL-17A, TNF-α, IL-1β, and IFN-γ or their infection with *Pseudomonas aeruginosa*, *Staphylococccus aureus*, *Staphylococcus epidermidis*, *Corynebacterium amycolatum, Escherichia coli*, *Enterococcus faecium* or the dermatophyte *Trichophyton rubrum* induced RNase 7 expression (reviewed in Becknell and Spencer, 2016; Rademacher et al., 2019). Keratinocyte infection with DENV was also shown to induce RNase 7 gene expression (Surasombatpattana et al., 2011).

The main characteristics of keratinocyte AMPs are summarized in Table 1.

**TABLE 1:** Main characteristics of the antimicrobial peptides synthesized by the keratinocyte.

AMP	Producing cells	Structure	Properties	References
LL-37	Keratinocytes, monocytes, mast cell granules, PMNs, natural killer (NK) cells, sweat glands	N-terminus signal peptide, cathelin domain, and C-terminus peptide	Antimicrobial, chemotaxis, cytokine/chemokine production, cell migration/proliferation	Frohm et al., 1997; Sørensen et al., 2001; Murakami et al., 2002; Di Nardo et al., 2003; Braff et al., 2005)
hBD1	Keratinocytes, monocytes, macrophages, DCs, sebaceous glands, canals of the sudoriparous glands	3 antiparallel beta sheets structure, and 3 disulfide bonds	Antimicrobial, chemotaxis, cytokine/chemokine production, wound healing, proinflammatory mediators/suppressors	Fulton et al., 1997; Ali et al., 2001; Semple and Dorin, 2012; Pace et al., 2017
hBD2	Keratinocytes, monocytes, macrophages, DCs			
hBD3	Keratinocytes			
hBD4	Keratinocytes			
S100A7	Keratinocytes	1 monomer consists of 5 helices each and carries only 1 calcium-binding EF-hand	Antimicrobial, chemotaxis, cytokine/chemokine production, wound healing, neutrophil migration, epithelial tumor progression marker	Gläser et al., 2005; Wolf et al., 2011
S100A8/9	Keratinocytes, macrophages, PMNs		Antimicrobial, chemotaxis, cytokine/chemokine production, antitumoral, antinociceptive	Ryckman et al., 2003
S100A15	Basal keratinocytes, melanocytes, DCs, LCs, sebocytes, smooth muscles and endothelial cells of the dermis		Antimicrobial, chemotaxis, cytokine/chemokine production, wound healing, neutrophil migration, epithelial tumor progression marker	Wolf et al., 2011; Hattinger et al., 2013
RNase 7	Keratinocytes	Hydrophobic signal peptide, mature peptide (12–16 kDa) with 3–4 disulfide bounds	Antimicrobial, immunomodulation	Becknell and Spencer, 2016; Rademacher et al., 2016

*AMP, Antimicrobial peptide; PMNs, polymorphonuclear neutrophils; DCs, dendritic cells; LCs, Langerhans cells.*

## Antiviral Activities of Keratinocyte AMPS

The antiviral activity of LL-37 has been reported against many viruses, both naked and enveloped as reviewed previously (Barlow et al., 2014; Ahmed et al., 2019a; Brice and Diamond, 2019). In particular, LL-37 was shown to inhibit viruses that replicate in the skin such as HSV-1 and -2, VZV, HHV-8, DENV, ZIKV, HPV16, or VACV (Howell et al., 2004; Buck et al., 2006; Hazrati et al., 2006; Crack et al., 2012; Alagarasu et al., 2017; Brice et al., 2018; He et al., 2018). Further antiviral activities were identified against viruses responsible for enteric infections such as Aichi virus A, respiratory diseases such as IAV, RSV and HRVs, and ocular epithelium infections such as adenoviruses (Gordon et al., 2005; Barlow et al., 2011; Uchio et al., 2013; Harcourt et al., 2016; Schögler et al., 2016; Findlay et al., 2017; Sousa et al., 2017; Vilas Boas et al., 2017). *In vitro* inhibition of HCV in hepatocyte-derived carcinoma HuH-7 cells and HIV in peripheral blood mononuclear cells (PBMCs) including CD4 + T cells was also described (Bergman et al., 2007; Matsumura et al., 2016). *In vivo*, the murine analog of LL-37, mCRAMP, reduced disease severity and IAV replication in the lung of infected mice to a similar extent as neuraminidase inhibitors (Barlow et al., 2011).

In the context of skin infections, antiviral properties of hBDs have been demonstrated against HSV, VZV, and VACV, similarly to LL-37, but also against EV-71 and Coxsackievirus (CV) A16, the main etiological agents of hand, foot and mouth disease (Hazrati et al., 2006; Howell et al., 2004; Crack et al., 2012; Chen et al., 2018). hBD-3, and in a lesser extent hBD-1, exerted anti-HSV-2 activities whereas hBD-2 did not but diminished VZV replication in HaCat cells, a keratinocyte cell line (Hazrati et al., 2006; Scudiero et al., 2010). Moreover, hBD-3, but not hBD-2, significantly reduced the expression of the VACV DNA-dependent RNA polymerase and the number of viral plaques in African green monkey kidney cell line BS-C-1 in a concentration-dependent manner from 5 μM (Howell et al., 2007). Finally, addition of recombinant hBD-3 to colon adenocarcinoma HT-29 cells inhibited EV-71, CV-A16, CV-B5, and poliovirus 1 infection. However, enterovirus replication was not impaired in genetically modified HT-29 cells overexpressing hBD-3 intracellularly, suggesting extracellular antiviral activity of the peptide (Chen et al., 2018). Regarding other viral species, hBD-1 and, more markedly, hBD-2 neutralized infectivity of the Phil82 strain of IAV (Doss et al., 2009). Antiviral activity of hBD-2 was also shown against RSV and type 3 human parainfluenza virus (HPIV-3) (Kota et al., 2008). Treatment of human lung epithelial A549 cells with 4 μg/mL hBD-2 reduced RSV and HPIV-3 viral titers by more than 100-fold whereas hBD-1 treatment had no effect against these two respiratory viruses (Kota et al., 2008). In addition, hBD-1 to -3 have been shown to render less infectious HIV-1 virion particles. Interestingly, this effect was higher when combining hBD-2 and hBD-3 than that of the peptides added separately (Quiñones-Mateu et al., 2003; Sun et al., 2005). Finally, hBD-1 to -4, used at 10, 20, and 50 μg/mL, diminished HCV gene expression and cytotoxicity associated with infection in PBMCs and HuH7.5 liver cell line (Mattar et al., 2016b). Conversely, other studies aimed at describing hBD antiviral properties found little or no activity. For example, hBD-1 and hBD-2 peptides had no effect on HPV16 infection of the cervical cancer cell line HeLa (Buck et al., 2006). However, polymorphisms in the gene DEFB1, encoding hBD-1, has been associated with higher susceptibility to HPV infection suggesting nevertheless a role for this peptide in the antiviral response. In the same way, BK and JC viruses were not or modestly inhibited by hBD-1 and hBD-2 while hBD-2 was found to be ineffective against HRVs (Dugan et al., 2008). Overall, the antiviral properties of hBDs are sometimes restricted to a given viral species suggesting a specific mechanism of action depending on the structure of the viral particle or its replication cycle. Finally, their ability to inhibit viral infection generally appears to be lower than that of LL-37 or other defensins.

To our knowledge, antiviral activity of psoriasin S100A7, calprotectin S100A8/9, and koebnerisin S100A15 has so far never been studied despite induction of their expression during many viral infections as described above. Similarly, RNase 7, despite its abundance in the skin, induction of its expression in an inflammatory or infectious context, and its high antimicrobial activity demonstrated *in vitro* against a broad spectrum of microorganisms such as Gram-positive and Gram-negative bacteria like *Pseudomonas aeruginosa*, *Staphylococcus aureus*, *Enterococcus faecium*, *Mycobacterium vaccae*, the yeast *Candida albicans* and *Pichia pastoris*, and the dermatophyte *Trichophyton rubrum*, has so far poorly been tested against viruses (Pulido et al., 2013; Rademacher et al., 2016). Recently, it was reported that RNase 7 failed to reduce HSV-1 infection in keratinocytes (Kopfnagel et al., 2020).

## Direct Antiviral Effects of Keratinocyte AMPS

AMPs can inhibit viral infection by targeting the steps preceding the entry of the virus into the cell but also intracellular stages of viral replication (Ahmed et al., 2019a). Before virus entry, they can directly alter viral particles by creating pores within the viral envelope thanks to their cationic and amphiphilic nature (Hsieh and Hartshorn, 2016). Electron microscopy observation of VACV and RSV, respectively exposed to LL-37 and hBD-2, thus showed a disruption of the viral envelope (Watson et al., 1998; Howell et al., 2004; Niyonsaba et al., 2005; Kota et al., 2008). LL-37-related inhibition of HHV-8 internalization in oral epithelial cells (OECs) relied on the same mechanism. The authors showed that LL-37 did not alter OECs, but, instead, the virions by disrupting the viral envelope then preventing viral entry into epithelial cells. This was observed from concentration of 10 μg/mL compatible with concentrations measured during inflammation in epithelial tissue reaching up to 20 μg/mL (Currie et al., 2013; Brice et al., 2018). Pre-incubation of ZIKV, IAV, VACV, and HCV with LL-37, or one of its analog, resulted in a significant decrease in the number of active virions suggesting, here again, an alteration of the viral particle (Ehrchen et al., 2009; Dean et al., 2010; Tripathi et al., 2013; Becknell and Spencer, 2016; Matsumura et al., 2016; Ulaeto et al., 2016; He et al., 2018). Similarly, hBDs, namely hBD-2, incubated with HIV, HPIV, and RSV also decreased virion infectivity, likely because of permeabilization of the viral envelope lipid bilayer since electron microscopy showed direct binding of hBDs to viral particles (Quiñones-Mateu et al., 2003; Kota et al., 2008). This detergent-like role is reported as the main AMP antiviral mechanism of action. However, direct interaction with AMPs can also cause viral particles extracellular aggregation blocking virus entry and leading to an increase of virus uptake by phagocytes. Treatment with LL-37 caused clumping of Venezuelan equine encephalitis virus (VEEV), thereby preventing cell infection (Ahmed et al., 2019b; Lai et al., 2011). Finally, the pre-fusion antiviral activity of AMPs can be linked to an inhibition of virus attachment to its receptor at the cell surface. LL-37 bound DENV-2 envelope protein acting as an entry inhibitor (Voss et al., 2011; Alagarasu et al., 2017). LL-37 also prevented HSV-1 infection in corneal epithelial cells by blocking viral-cell attachment (Sunahori et al., 2006; Lee et al., 2014). hBD-3 interacted with either the HSV receptor at the target cell surface or the HSV glycoprotein on the viral envelope, thereby eliciting a stronger inhibition of viral entry (Hazrati et al., 2006; Niyonsaba et al., 2008).

In addition to these antiviral properties based on the inhibition of the virus entry into the target cell, AMPs can interfere with intracellular steps of viral replication (Hazrati et al., 2006; Niyonsaba et al., 2008; Wilson et al., 2013). Indeed, several studies reported that AMPs added after the virus entry could lead to virus gene expression or genome replication inhibition (Howell et al., 2006; Crack et al., 2012; Currie et al., 2013; Sousa et al., 2017). Moreover, the hypothesis of intracellular antiviral activity of AMPs is supported by the fact that recombinant peptides added to the cell culture medium can be internalized by the epithelial cells (Lau et al., 2005). Unfortunately, most of the time, the exact mechanism of the post-fusion antiviral activity of AMPs is not clearly defined. An elegant study nonetheless described the anti-HIV-1 intracellular activity of LL-37 that occurs through direct protein-protein interactions with reverse transcriptase and, in a lesser extent, protease (Wong et al., 2011). In contrast, the LL-37 did not have the ability to prevent the translocation of HIV-1 integrase from the cytoplasm into the nucleus, which is its site of action. Furthermore, inhibition of early HIV-1 transcription by hBD-2 has also been reported (Klotman and Chang, 2006; Yong and Luo, 2018). Overall, the direct mechanisms by which AMPs inhibit virus infection remain little known, particularly regarding the intracellular steps of replication. The immunomodulatory functions of AMPs were, however, better studied, although much progress remains to be made in the context of viral infection.

## Indirect Antiviral Activities Through Modulation of the Host Cell Immune Response

### Induction of Cytokine and Chemokine Expression by Keratinocyte AMPs

The expression of antimicrobial peptides synthesized by the keratinocyte is increased or induced in context of inflammation and/or infection. In turn, these peptides can stimulate the expression of cytokines and chemokines because of their own pro-inflammatory properties or by their capacity to potentiate an already in progress inflammatory response.

#### Intrinsic Pro-inflammatory Properties of AMPs

AMPs are known to have intrinsic pro-inflammatory properties through induction of various inflammatory mediator production by resident skin cells, such as keratinocytes, and cutaneous immune cells such as PBMCs and PMNs. AMPs act by binding cellular receptors leading to signaling pathway activation and up-regulation of cytokine or chemokine expression. CC Chemokine Receptor 6 (CCR6), TLR4, and G Protein-Coupled Receptor (GPCR) are the three receptors identified so far with which AMPs interact to induce the cellular inflammatory response.

CCR6 is a seven-transmembrane domain G-protein-coupled receptor with only one known chemokine ligand, CCL-20, which was involved in DC, memory T cell and selected B cell subtype chemotaxis (Lee and Körner, 2017). hDB-3 binding to CCR6 upregulated IL-37 expression and release by human keratinocytes through caspase-1 and -4, mothers against decapentaplegic homolog 3 (SMAD3), mitogen activated protein kinase (MAPK) and nuclear factor-kappa B (NF-κB) pathway activation (Smithrithee et al., 2015).

**FIGURE 1:**
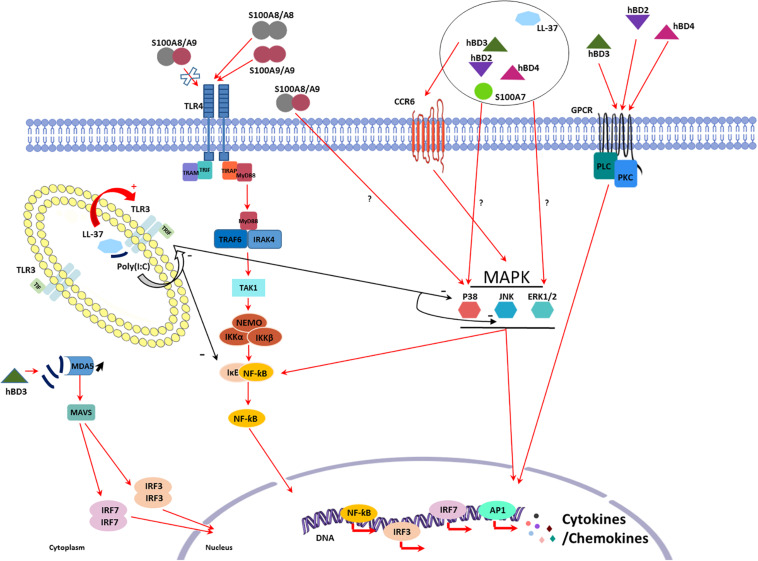
Schematic representation of the known mechanisms involved in the intrinsic proinflammatory properties of LL-37, human β-defensins (hBDs), and peptides from the S100 family. CC Chemokine Receptor 6 (CCR6), Toll-like receptor (TLR)4, and G Protein-Coupled Receptor (GPCR) are the three main receptors identified for antimicrobial peptides (AMPs) at the cell surface. AMPs exert direct proinflammatory effects downstream receptor binding through activation of several signaling pathways involving p38 mitogen-activated protein kinases (MAPK), extracellular signal-regulated kinases (ERK), nuclear factor-kappa B (NF-κB), and phospholipase C (PLC) leading to cytokine and chemokine production. Some pro-inflammatory effects have also been described involving p38 and ERK signaling pathways without identification of the receptor involved. AMPs can finally modulate pathogen associated molecular patterns (PAMPs) inflammatory response as seen with poly(I:C). When co-administrated with poly(I:C), hBD3 lead to an increase in IFNβ production by reducing the poly(I:C) uptake in endosome and thus increasing signaling through MDA5. LL-37 and poly(I:C) form a complex that can enhance or decrease TLR3 signaling.

TLR4 normally senses bacterial peptidoglycan and lipopeptides as well as viral envelop glycoproteins. Its interaction with S100A8 and S100A9 homodimers induced IL-1β, IL-6, INF-γ, and TNF-α secretion in human PBMCs. Interestingly, S100A8/S100A9 heterodimer binding to TLR4 failed to induce this secretion (Chen et al., 2015). Furthermore, treatment with the recombinant S100A8-GST peptide stimulated macrophages, again through TLR4 activation, increasing TNF-α, CCL-2, IL-1β, IL-6, IL-12, IL-22, IL-23, and IL-24 mRNA expression and contributing to their migration (Figure 1; Inoue et al., 2018).

G protein-coupled receptor (GPCR) and phospholipase C (PLC) signaling pathway are involved in hBD-2, -3, and -4-mediated induction of IL-6, IL-10, IFN-γ, CXL-10, CCL-2, and CCL-5 expression and secretion in human primary keratinocytes playing a role in their migration and proliferation (Figure 1; Niyonsaba et al., 2007).

Other signaling pathways have been described activated by the PAMPs expressed by keratinocytes even if the receptor involved has not always been identified. Niyonsaba et al. (2005) demonstrated that IL-18 mRNA expression and IL-18 secretion were induced by stimulation of keratinocytes with hBD-2, -3, -4, but not -1, and LL-37 through the phosphorylation of p38 and ERK1/2. Moreover, the involvement of p38 and ERK1/2 was also described in IL-6, CXCL-8, CCL-3, CCL-20, TNF-α, and ROS production a dose- and time-dependent manner after human PMN treatment with S100A7 (Zheng et al., 2008). Similar results were obtained by addition of S100A7 to keratinocytes that increased production of CXCL-8, CXCL-10, and CCL20 (Niyonsaba et al., 2008). hBD-2 and, in a lesser extent, hBD-1 and hBD-3 were shown to induce expression and secretion of IL-6, IL-10, and CXCL-8 in PBMCs in a dose-dependent manner (Boniotto et al., 2006). hBD-3 can also stimulate the expression of IL-1α, IL-6, CXCL-8, and CCL-18 in differentiated macrophages as well as CXCL-1, CCL-2, CCL-22, MIP-1α, IL-1β, and VEGF in monocytes (Jin et al., 2010; Petrov et al., 2013). hBD-2 also causes pro-inflammatory cytokine secretion by lymphocytes through activation of JNK, MERK/ERK and PI3K/Akt pathways (Kanda et al., 2011). Concerning RNase 7, its ability to induce IFN-α and IFN-stimulated gene expression in human plasmacytoid DCs and PBMCs has been reported (Kopfnagel et al., 2018). Finally, TNF-α, IL-1β, IL-6, and CXCL-8 production observed in S100A8/A9-stimulated monocytes involved both p38 MAPK and NF-κB signaling pathway activation in an independent manner (Figure 1; Sunahori et al., 2006).

#### Modulation of Inflammatory Response by AMPs

Besides leading to cytokine and chemokine expression and secretion, AMPs can also contribute to enhance or inhibit inflammatory response induced by PAMPs.

Polyinosinic:polycytidylic acid [poly (I:C)] is a synthetic analog of double stranded RNA, mimicking a molecular pattern associated with viral replication known to activate TLR3, RIG-I, and MDA5. LL-37 was shown to increase IFNβ-1 mRNA expression induced by poly (I:C) stimulation in human epidermal keratinocytes leading to an enhanced antiviral activity against HSV-1 (Takiguchi et al., 2014; Sato et al., 2018). Lai et al. (2011) demonstrated that this LL-37-dependent enhancement of the inflammatory response required TLR3. Indeed, LL-37 forms, with poly (I:C), a complex that enhances TLR3 signaling pathway (Singh et al., 2013). In contrast, the innate immune response induced by poly (I:C) in macrophages was inhibited by high concentrations of LL-37 (5 μM) resulting in a decreased TNF-α and nitrite production as well as IL-1β and IL-6 mRNA expression (Hasan et al., 2011). This apparently paradoxical effect could be due to the capacity of LL-37 to inhibit phosphorylation of Iκb, MAPKs p38, and JNK induced by poly (I:C) in macrophages (Hasan et al., 2011). Thus, the LL-37-poly (I:C) complex could either prevent TLR3 activation or potentiate TLR3-dependant signaling (Singh et al., 2013).

Modulation of the innate immune response to TLR agonists was also demonstrated with hBD-3. Semple et al. (2015) observed an increased production of IFN-β, TNF-α, CXCL-8, and IL-6 in monocytes and PBMCs stimulated with poly (I:C) in presence of hBD-3. Higher levels of IFN-β and TNF-α were also observed in transgenic mice expressing hBD-3 and stimulated with poly (I:C) as compared to control mice (Semple et al., 2015). In contrast to LL-37, hBD-3 doesn’t form a complex with poly (I:C) to modulate its effects since the two molecules do not co-localize. However, hBD-3 altered poly (I:C) localization within the cell cytoplasm since, in the presence of HBD-3, less poly (I:C) localized to the early endosome. The authors demonstrated that hBD-3 suppressed the poly (I:C)-induced TLR3 response mediated by TIR-domain-containing adapter-inducing interferon-β (TRIF), while exacerbating the cytoplasmic response through MDA5 and MAVS (Vazquez and Horner, 2015).

In other hand, TLR9-mediated DNA sensing was strongly enhanced in presence of RNase 7 leading to secretion of antiviral level of IFN-α by human plasmacytoid DCs (Kopfnagel et al., 2018). Among the known immunomodulatory activities of RNase 7 (reviewed in Rademacher et al., 2019), it was shown that RNase 7 can contribute to antiviral immune response of human keratinocytes through promotion of self-DNA sensing (Kopfnagel et al., 2020). Indeed, pretreatment with both RNase 7 and DNA reduced HSV-1 replication in keratinocyte, a phenomenon mediated by induction of IFN-β production (Kopfnagel et al., 2020).

AMPs are also able to modulate inflammatory response induced by viral infection. The addition of LL-37 to HRV-infected human bronchial epithelial cells enhanced IL-6 and CCL-2 production (Lai et al., 2011). It also increased the expression of type I IFN during VEEV infection (Lai et al., 2011; Ahmed et al., 2019b). However, paradoxical pro- and anti-inflammatory properties of LL-37 were also observed in the context of viral infection (Tripathi et al., 2014). In one hand, CXCL-8 production induced by PMN infection with IAV was reduced in cell supernatant in presence of LL-37, while on the other, LL-37 enhanced PMN extracellular traps (NETs) formation and stimulated respiratory oxidative bursts in IAV-infected PMN (Tripathi et al., 2014). It is interesting to note that the anti-IAV mechanism of LL-37 through PMN activation was different from that reported for hNPs and hBD-2, which promoted virus aggregation and then phagocyte uptake by PMNs (Tecle et al., 2007). hBD-2 had also the ability to stimulate antiviral immunity both *in vitro* and *in vivo* (Kim et al., 2018). When conjugated with the receptor-binding domain of Middle East respiratory syndrome-coronavirus spike protein (S-RBD), it significantly increased the expression levels of IFNs, IFN-stimulated genes and chemokines capable of recruiting leukocytes including macrophages, T cells, and DCs at the site of infection. *In vivo*, immunization of mice with hBD-2-conjugated S-RBD enhanced the immunogenicity of the S-RBD and elicited a higher S-RBD-specific neutralizing antibody response than S-RBD alone. Finally, hBD-4 may also enhance antiviral host protection. Administration of recombinant murine hBD-4 into animals immediately prior to IAV infection resulted in a significant increase of IFN-γ concentration in bronchoalveolar lavage (LeMessurier et al., 2016).

### Chemotaxis and Immune Cell Activation

In addition to cytokine and chemokine production modulation, keratinocyte AMPs can also modify the innate immune cell profile at the site of infection and inflammatory response. They can modulate the cellular composition of the inflammatory infiltrate but also the state of maturation and activation of the infiltrating cells.

#### Chemotactic Properties of Keratinocyte AMPs

**FIGURE 2:**
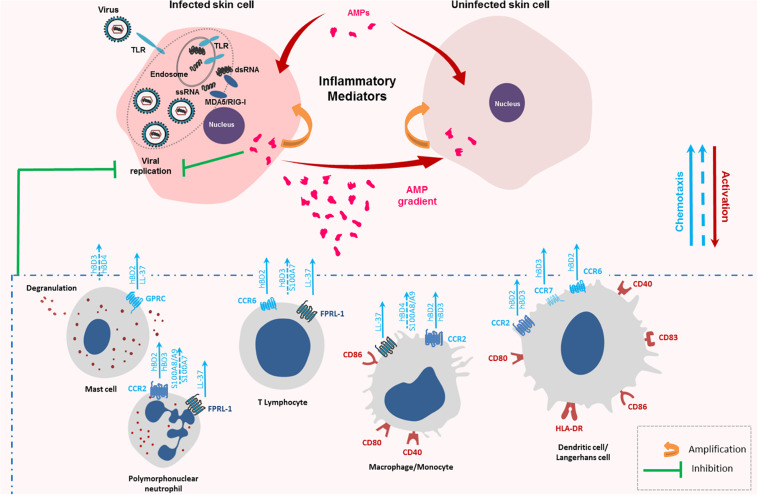
Chemotactic and immune cell activation properties of keratinocyte antimicrobial peptides (AMPs). Keratinocytes infected with RNA viruses, such as arboviruses, sense viral single-stranded and double-stranded RNA generating an innate immune response made of cytokines, chemokines and AMPs. AMPs can then trigger the inflammatory response of the infected cells as well as non-infected cells. They also attract a wide range of immune cells at the site of the infection through several receptors as CC Chemokine Receptor (CCR)-2, CCR-6, CCR-7, and Formyl Peptide Receptor-Like (FPRL)-1 contributing to enhance the innate immune response and initiate the adaptative one. These receptor-dependent chemoattractant effects are represented with full blue arrows. Chemotactic effects described without identification of the AMP receptor are represented by a discontinuous blue arrow.

AMPs can attract immune cells at the site of infection promoting an inflammatory context favorable to pathogen eradication. LL-37, hBD-2, -3, and -4 chemotactic activity has been observed on PMNs, T cells and monocytes (Figure 2; De et al., 2000; García et al., 2001; Röhrl et al., 2010). For hBDs, the mechanism was mediated through binding to the chemokine receptor CCR2 attracting CCR2-expressing inflammatory cells such as monocytes/macrophages, DCs, and PMNs to the sites of infection while LL-37 chemotactic activity was mediated by the G protein-coupled formyl peptide receptor-like 1 (FRPL-1) (De et al., 2000; Jia et al., 2008; Lin et al., 2008; Röhrl et al., 2010). *In vivo*, injection of hBD-2 in mice peritoneal cavity induced macrophage migration, a mechanism shown to be independent of the CCR6 receptor (Soruri et al., 2007). Other studies demonstrated an AMP-related chemotaxis on mast cells (Niyonsaba et al., 2002; Soruri et al., 2007). While hBD-2,-and LL-37 were shown to act as a specific mast cell chemotaxin through activation of G-protein-PLC-sensitive signaling pathway (Figure 2; Chen et al., 2007; Soruri et al., 2007), hBD3 and -4 were involved in mast-cell chemotaxis through MAPK pathway activation (ERK, JNK, and p38 phosphorylation) (Soruri et al., 2007). Finally, hBD-2-induced chemoattraction was also observed with immature DCs and memory T cells through CCR6 binding, while hBD-3, after CCR7 binding, promoted migration and lymph node localization of treated LC-DCs (Figure 2; Yang et al., 1999). Otherwise, S100 peptides may also display chemotactic activity. S100A7 was shown as a potent and selective chemotactic protein for CD4 + T lymphocytes and PMNs but had no effect on monocytes (Jinquan et al., 1996). Chemotactic effect of the S100A8/A9 heterodimer was observed with macrophages and PMNs (Figure 2; Ryckman et al., 2003; Hiratsuka et al., 2008; Chen et al., 2015). Taken together, these data suggest that AMPs can favor the migration of immune cells crucial for mounting successful immune responses against viruses.

#### Immune Cell Activation by Keratinocyte AMPs

In addition to chemotaxis, keratinocyte AMPs can cause maturation and activation of innate immune cells. LL-37 enhanced CD86, CD83, and CCR7 expression on the surface of murine LCs indicating cell maturation (Figure 2; Ogawa et al., 2013). Moreover, incubation with hBD-3 resulted in monocyte and myeloid DCs maturation revealed by CD80, CD86, and CD40 upregulation (Figure 2). This was not observed with plasmacytoid DCs or B lymphocytes. MyD88 was involved in this maturation suggesting a TLR4-mediated process (Funderburg et al., 2007). Another study confirmed induction of phenotypic LC-DCs maturation by hBD-3 (Ferris et al., 2013). In the presence of the peptide, an increase in HLA-DR, CD83, CD86, and CCR7 expression on human immature LCs and DCs was assessed (Figure 2). These data also demonstrated that hBD-3 exposure allowed potent antigen presentation capacity in LC-DCs and high levels of IFN-γ production by primed T-cells suggesting that the peptide skewed cell activation toward a Th1-type immune response (Ferris et al., 2013). In addition to the binding to CCR6 and CCR2, hBD-3 was shown to interact with TLR1 and TLR2 on antigen-presenting cells, such as myeloid dendritic cells, leading to their activation (Funderburg et al., 2007). This peptide also induced the monocytes costimulatory molecules, CD80 and CD86, necessary for T cell activation (Figure 2; Petrov et al., 2013). Finally, Chen et al. (2007) demonstrated the abilities of hBD-3 and -4 to cause mast cell degranulation, prostaglandine D2 production and chemotaxis (Figure 2). Thus, AMPs, through their immune cell activation ability, can contribute to stimulate innate immunity and activate lymphocytes, key components of adaptive immunity against viruses.

## Other Cell Modification Caused by Keratinocyte AMPS Impacting Viral Infection

### Induction of APOBEC Expression

Cell signaling pathways mediated by the chemokine receptor CCR6 also play a role in defensin-mediated HIV replication inhibition. hBD-2 treatment was shown to up-regulate expression of host restriction factor apolipoprotein B mRNA-editing enzyme-catalytic polypeptide-like 3G (APOBEC3G) in PBMCs or CD4 + T cells. APOBEC3G is an HIV-1 restriction factor that inhibits the accumulation of early reverse transcription products during virus replication cycle (Wilson et al., 2013). This induction was mediated through the activation of CCR6 by hBD-2 (Lafferty et al., 2010).

### Modulation of Chemokine Receptor Expression at the Surface of the Target Cell

Chemokine receptors CXCR4 and CCR5 are HIV-1 co-receptors in addition to CD4. The HIV-1 strains use either of these two co-receptors to infect CD4 + cells. hBD-2 and -3 reduced cell surface expression of CXCR4, but not CCR5, in PBMCs and a human T cell line (Quiñones-Mateu et al., 2003; Feng et al., 2006). Those peptides, by modulating host surface receptors expression, acted as antiviral compound restricting cell binding and entry of HIV-1 strain with CXCR4 tropism (Quiñones-Mateu et al., 2003; Feng et al., 2006).

Conversely, LL-37 enhanced HIV-1 infection of monocyte-derived Langerhans cells (mLCs) (Ogawa et al., 2013). LL-37 treatment increased CCR5 and CD4 expression on mLCs surface, which could explain the potentiating effect of LL-37 on HIV-1 infection. Effects of the peptide were also studied in DCs. Inhibition of HIV-1 infection was observed in LL-37 treated DCs, a phenomenon which may be due to down regulation of DC-SIGN and/or CCR5 expression (Ogawa et al., 2013). Finally, LL-37 facilitated HIV-1 transmission from mLCs to CD4 + T cells whereas opposite effect was observed using DCs (Ogawa et al., 2013).

## Conclusion

The keratinocyte is the target cell of many viruses of major importance in human health. As an immune cell that can detect viral PAMPs, it has the ability to secrete a wide range of molecules in response to the infection including many antimicrobial peptides. These peptides may then act directly on the viral particle or its replication cycle as well as modulate the innate immune response of the host. The first objective of this immunomodulation is likely to create an antiviral state by potentiating the production of cytokines and chemokines, and attracting immune cells to the site of infection. However, its precise role in the pathophysiology of viral infection remains to be defined. Furthermore, this dual mode of direct and indirect antiviral action suggests that AMPs may have a promising therapeutic role with direct virucidal activity and limited risk of engendering viral resistance. The deployment of such potentially impactful, innovative and reliable compounds awaits success in future research on how to effectively administer them and possibly stimulate them *in situ*.

## Author Contributions

CC and MG wrote the draft. CB, CJ, and NL contributed to the revision of the manuscript. MW designed the figures. All authors approved the final version of the manuscript.

## Conflict of Interest

The authors declare that the research was conducted in the absence of any commercial or financial relationships that could be construed as a potential conflict of interest.
